# The Cost Implication of Trastuzumab in Patients With HER2-Positive Breast Cancer at Moi Teaching and Referal Hospital, Eldoret, Kenya

**DOI:** 10.1200/GO.22.54000

**Published:** 2022-05-05

**Authors:** Lucy Najala Wabende, Manisha Bhatia, Stephen Kiptoo, Nicholas Kisilu, Caroline Kiboss, Silvanus Kibiwot, Sally Jepkirui, Dorice Adhiambo Awuor, Naftali Busakhala, Joanna Hunter-Squires

**Affiliations:** ^1^ Academic Model Providing Access to Healthcare—Moi Teaching and Referral Hospital, Eldoret, Kenya; ^2^ Indiana University, Bloomington, IN; ^3^ Moi University, School of Medicine, Eldoret, Kenya

## Abstract

A year course of trastuzumab revolutionized treatment for human epidermal growth factor receptor (HER2)-positive breast cancer by increasing survival rates. However, trastuzumab treatment is cost-prohibitive, and data on abbreviated courses is lacking in advanced disease. At Moi Teaching and Referral Hospital (MTRH), one of two national referral hospitals in Kenya, HER2-positive breast cancer accounts for 20% and 95% of patients present with Stage III/IV disease. The national health insurance fund (NHIF) covers four of the recommended 18 cycles. In this study, we aimed to understand varying trastuzumab treatment plans and patient adherence.

**METHODS:**

This is a retrospective chart review for breast cancer patients seen at the MTRH oncology clinic from January to December 2020. Immunohistochemistry results were reviewed and those with HER2+ by immunohistochemistry were included. Data was analyzed via NVivo with multivariate analysis.

**RESULTS:**

Ninety-five patients were included with an average age of 48.7 years. 33.59% (n = 31) completed 18 cycles of trastuzumab. However, 53.3% (n = 16) of those who completed the treatment were inconsistent in treatment schedule. Nearly half, 45.26% (n = 43), received only the four cycles being covered by NHIF. More concerning, 23.15% (n = 22) did not begin trastuzumab. For MTRH patients, the cost of trastuzumab is 111,244 Kenyan Shillings (990 USD), 9 times the average monthly income per family in western Kenya.

**CONCLUSION:**

Although trastuzumab is lifesaving, < 33% of patients with HER2-positive breast cancer complete treatment in the public sector in western Kenya. Of these patients, half experience treatment delays that may be attributable to secondary costs related to travel and absence from employment. External charity funding has improved patient access but is unsustainable. The Kenyan Ministry of Health can reduce the cost burden for patients by negotiating with pharmaceutical companies to offer subsidies or adjusting NHIF policies to promote drug access. Further studies into shorter course trastuzumab are merited.

